# A Comprehensive Dataset of Factory Farms in California Compiled Using Computer Vision and Human Validation

**DOI:** 10.1038/s41597-025-06082-6

**Published:** 2025-11-19

**Authors:** Varun Magesh, Nicolas Rothbacher, Saskia Comess, Erin Maneri, Kit Rodolfa, Sara Tartof, Joan Casey, Keeve Nachman, Daniel E. Ho

**Affiliations:** 1 Stanford Regulation, Evaluation and Governance Lab, Stanford Law School, Stanford University, Stanford, CA USA; 2 Emmett Interdisciplinary Program in Environment and Resources, Stanford University, Stanford, CA USA; 3 Department of Research and Evaluation, Kaiser Permanente of Southern California, Pasadena, CA USA; 4 Department of Environmental and Occupational Health Sciences, University of Washington, Seattle, WA USA; 5 Department of Environmental Health and Engineering, Johns Hopkins Bloomberg School of Public Health, Baltimore, MD USA

**Keywords:** Environmental impact, Agriculture

## Abstract

Concentrated Animal Feeding Operations (CAFOs) house livestock at high densities for prolonged periods of time, posing substantial risks to environmental and human health. However, limited public information on CAFOs has constrained efforts to quantify their impacts on proximate communities. Gaps in permitting and reporting have severely limited studies that rely primarily on administrative records. We introduce Cal-FF, a near-complete census of CAFOs in California, a large and agriculturally significant state in the United States, with richer facility data than existing administrative data. Cal-FF was constructed using computer vision on satellite imagery, along with extensive human validation. We focus on California, which accounts for about 20% of US livestock production and has been at the forefront of CAFO regulatory innovation. We estimate that our 2,121 facility dataset captures 98% (95% CI [82%, 98%]) of all California CAFOs as of 2017, identifying 222 locations not present in state regulatory records. In addition to improved accuracy, Cal-FF adds a wealth of information about each facility, including validated permit information, land parcel data, satellite imagery, and annotated facility features. These data provide numerous opportunities for research, analysis, and monitoring.

## Background & Summary

Modern reliance on Concentrated Animal Feeding Operations (CAFOs) causes harm to water, air, land, and climate. Concentrated production of large amounts of animal waste (manure, urine, and bedding material) can lead to air and water pollution in surrounding areas^1,2^. Considering just ammonia and phosphorous pollution in surface water, researchers estimate that each marginal CAFO results in water quality damages in excess of $200,000 per year^3^. Agriculture represents the largest source of anthropogenic methane, a potent greenhouse gas, in the US^4^. Enteric fermentation by ruminant animals (e.g. cattle) and manure management contribute roughly 30% of these total methane emissions, exceeding the contributions of natural gas and landfills^5^. Researchers attempting to understand and mitigate the environmental impacts of CAFOs have been hindered by unreliable and incomplete data about the true number of CAFOs, their locations, and their characteristics^6^. Even regulators responsible for monitoring CAFOs face similar problems; reporting requirements and awareness of CAFO locations and activities remain insufficient^2,7^. Attempts to incentivize and strengthen permitting and enforcement have been largely unsuccessful or rolled back by legal challenges^7^. Instead, efforts to study the consequences of factory farming operations have relied on regional estimates^8^, land-use data^9^, or machine-learning predictions^10^. Accurate, precise, and rich data are necessary to understand how CAFOs affect neighboring populations and to understand the facility-level impact of policies intended to mitigate these effects. This work provides such data for CAFOs in the state of California.

Estimates show that California has one of the largest number of CAFO operations in the country^11^ and has more than 1.7 million dairy cattle^12^. The state is also a pioneer in policies to limit pollution and uphold animal welfare standards^13^, presenting researchers with an opportunity to evaluate the impact of novel regulatory approaches. Despite this, state regulatory data is inconsistent and unreliable. Nine different Regional Water Quality boards implement federal and state water laws, but regulation and enforcement can vary by region as long as certain minimum standards are met^14–16^. The permit data itself is rife with missingness and lacks consistency.

For example, a researcher relying on California’s permit records would find a list of 3,761 permits — far more than the number of CAFOs estimated to be in the state by the USDA^17,18^. The researcher might try to filter on the reported ‘Animal Population’ field — only to find that nearly 1 in 4 permits either report no animal population number or zero animals. 420 permits do not report a location; 20 list their address only as ‘CA’; 676 addresses appear more than once; and the permit’s registered location may match a parcel containing a CAFO only 40% of the time. Researchers cannot be sure if they have captured all (or even most) facilities of interest — California’s permit compliance rate is unknown. In short, granular analysis of the environmental and health impacts of California CAFOs is severely constrained by available administrative data.

We respond to this shortcoming by developing a rich and comprehensive near-census of California CAFOs. We adapt recent advances in satellite-based CAFO detection models^19,20^ to livestock facilities in the state of California, which differ from facilities in other regions (see for example Supplemental Figure S1 comparing CAFOs in California and the DelMarVa region). We use these detections as a starting point, followed by a review of all candidate facilities detected by our model. For each candidate facility, we confirm that it is a CAFO and enrich its location with extensive annotation, including the facility’s geographic footprint, animal type, and construction information. Note that regulatory definitions of the term “CAFO” can vary by jurisdiction and generally rely on animal count thresholds. In the present work, we do not have detailed information on animal count; we use the term “CAFO” more generally to refer to large-scale livestock operations that meet the criteria described in the Methods section. We also measure the *completeness* of the dataset (the percentage of all CAFOs in California that we capture) by reviewing a stratified sample of imagery without detections. We confirm that our dataset includes an estimated 98% (95% confidence interval [82%, 98%]) of all facilities in California, very near a complete population enumeration.

**Fig. 1:**
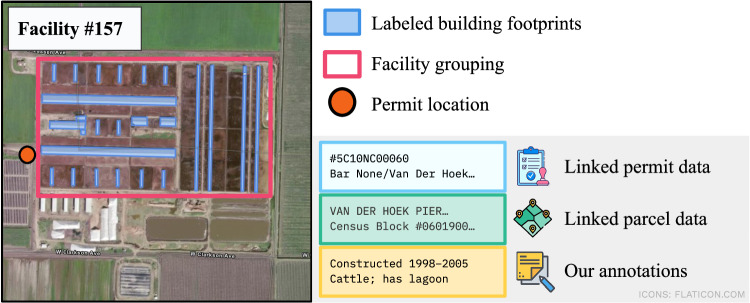
A representative facility in our dataset. This figure shows the extensive data we have compiled for each facility, including a range of geographic data, structured data from different sources, and our own annotations on facility features.

The resulting dataset contains 24,818 buildings aggregated into 2,121 facilities (an example location is shown in Fig. 1). These facilities are concentrated in California’s Central Valley, as seen in Table 1, which summarizes facilities by county, including a count of facilities that are unreported in administrative data. Figure 1 shows the details included in our dataset, including permit data, parcel data, construction timelines, and annotated animal types, lagoon information, and more. The richness of the dataset enabled investigations and inquiries not previously possible with limited and unreliable permit data. Analyses relying solely on registered locations in administrative records miss at least 10.5% facilities that we report, and provide misleading or inaccurate location information on around 45.0% of facilities. Understanding the existence and location of un- and under-permitted facilities allows researchers to investigate the risk of these facilities on their surrounding communities.

**Table 1:** An overview of our data and the issues present in the state permit data.

County	Facilities		Permits			
	Total	No Permit <1km	Total	No Facility <1km	>200 Animals	No Animal Count
Tulare	356	15	448	20	400	41
Merced	313	15	443	64	333	82
Fresno	160	15	211	32	149	54
Stanislaus	322	12	428	33	304	82
Madera	68	12	69	11	52	15
San Joaquin	156	11	216	22	156	42
San Diego	21	11	30	16	17	7
Kings	188	8	305	13	236	50
Sonoma	86	7	151	17	96	26
Riverside	41	7	292	191	149	137
Imperial	32	5	34	6	31	3
Kern	67	5	97	6	85	9
San Bernardino	82	4	601	123	316	243
Humboldt	65	2	120	13	66	21
All Other	164	28	311	119	145	97
Total	2121	157	3756	686	2535	909

First, the number of CAFOs we label in the state, separated by county and the number of facilities that do not have a permit anywhere within 1 km. Next, the number of permits present in each county and the number without a labeled CAFO within 1 km. Finally, the number of permits in each county that have enough animals present to possibly be considered a CAFO and the number of permits that do not report any animal count. Permit information does not accurately capture facility presence: a portion of facilities and permits are not co-located and a large proportion of permits do not report any animal count information.

Cal-FF provides the most comprehensive view to date of CAFOs in California, including curated metadata on animal types, detailed permit information, and historical construction and destruction timeframes. This work will enable robust investigations into important questions regarding the role of CAFOs in environmental pollution, community exposure, and public health, opening avenues of research that would not otherwise be possible.

## Methods

**Fig. 2:**
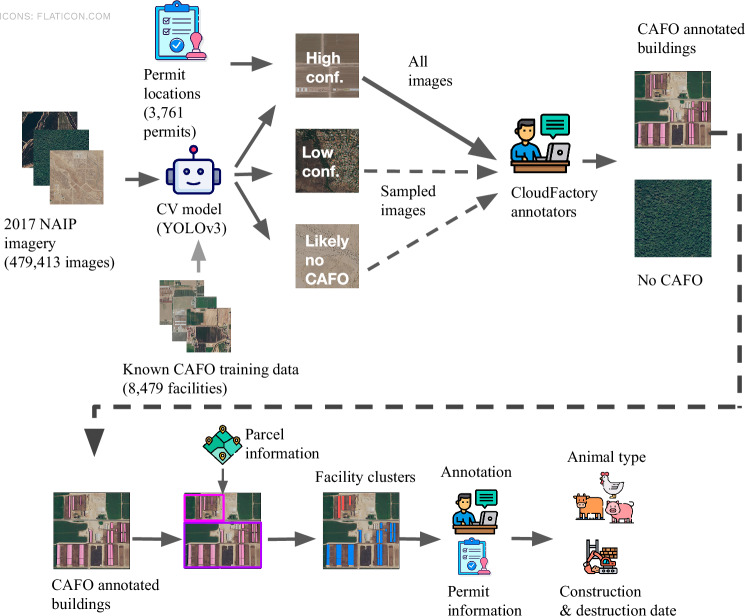
Dataset creation pipeline. We use multiple rounds of human labeling and several sources of data to accurately identify CAFO facilities and information about their animal types and construction dates.

Our methods for mapping CAFOs in California combine computer vision modeling techniques with extensive image annotation and metadata collation to create an accurate, complete, and rich dataset. We allocate our human labeling efforts by training and using a computer vision model to stratify images in the state based on their likelihood of containing a CAFO, allowing us to efficiently annotate a nearly comprehensive set of CAFOs in the state while at the same time developing a robust estimate of the total population of facilities to confirm our dataset’s high degree of coverage. This is a significant extension beyond other methods applied to CAFO mapping: previous datasets only report model predictions^20^ (which are helpful, but not entirely complete or precise) or annotated administrative records^21^ (which do not include unpermitted facilities). We augment our dataset of building locations by developing clustering methods to group annotated buildings into facilities, which are the regulated unit in California, and further annotate these facilities with construction and destruction dates, animal types, and finally, permit records and parcel ownership information. An overview of our approach is shown in Fig. 2.

### CAFO Definition

US EPA defines Animal Feeding Operations (AFOs) as “agricultural operations where animals are kept and raised in confined situations”; this definition specifically hinges on the duration of confinement (animals are confined for at least 45 days in a 12-month period) and source of animal feed (crops/vegetation are not sustained on the facility property). The EPA differentiates AFOs and CAFOs by the number of animals housed^22^ and defines a ‘Medium CAFO’ as one that houses more than 200 dairy cows or 300 cattle and a ‘Large CAFO’ as one that houses more than 700 dairy cows or 1,000 cattle. These animal-count based definitions are challenging to implement in satellite surveys because there is little ground truth data about the number of animals housed in facilities. Outdoor animal housing is also common in California, making it difficult to estimate facility animal count based on size.

**Fig. 3:**
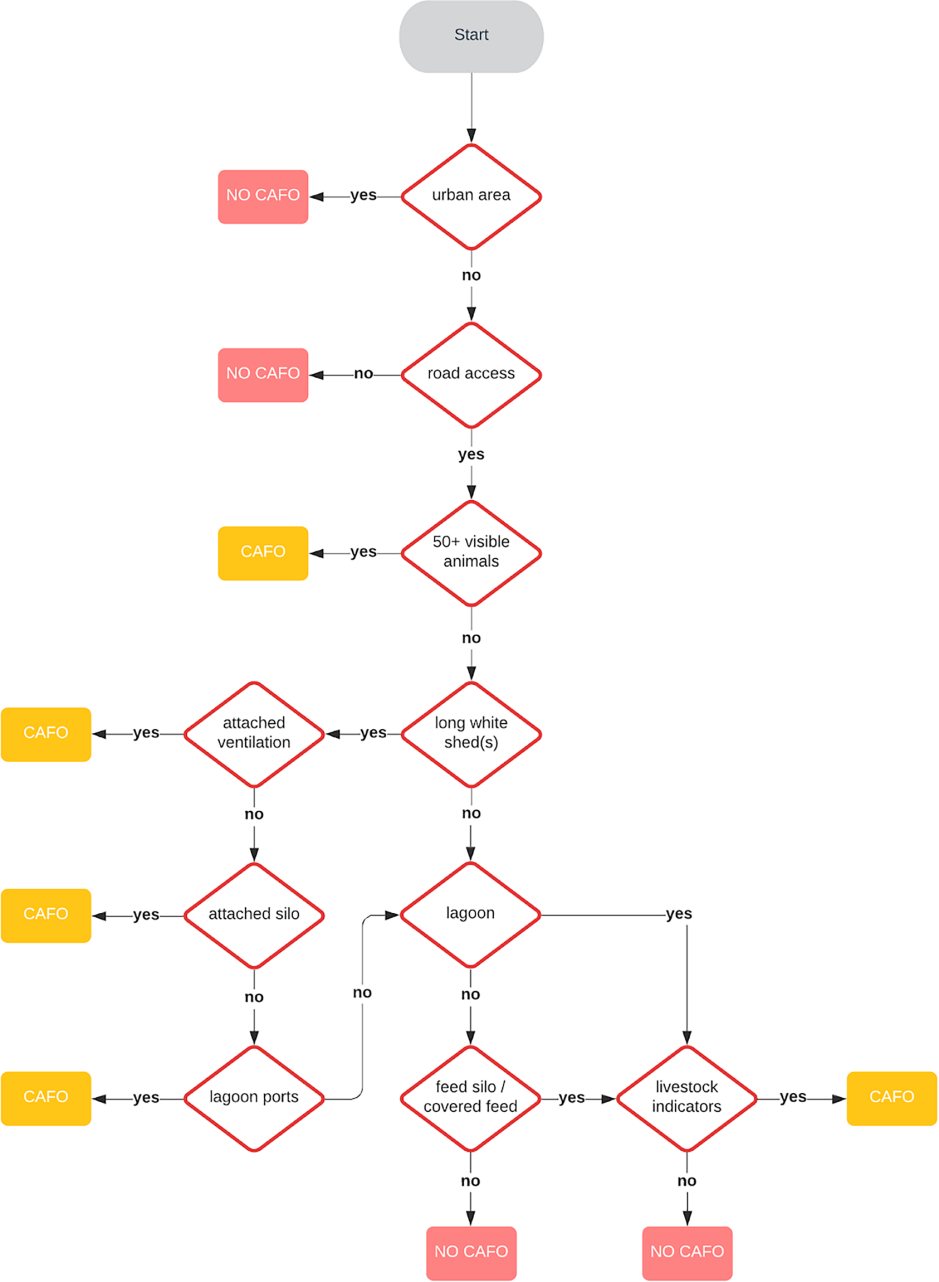
Guidelines used by humans to decide whether a building should be annotated as a part of a CAFO.

For the purposes of this work, we use visual evidence of large-scale animal housing as the key physical indicator for CAFOs. Infrastructure dedicated to housing large numbers of animals — like wastewater lagoons, ventilation ports, and large scale animal feed storage — indicate the presence of many animals and potential for environmental impact. While this definition is not a perfect match for regulatory definitions of CAFOs, it is a practical way to identify facilities that are likely to have a significant environmental impact and are of interest to regulators and researchers. We use this definition to guide our human labelers; an example of the labeling guidelines is shown in Fig. 3. Applying this definition requires some judgment, but we find that annotators have a strong degree of agreement (with a Cohen’s kappa of 0.73) on individual CAFO labels. The supplementary materials engage in a longer discussion of measurement consistency and inter-rater reliability. We present Cal-FF and population estimates based on the result of three different rounds of individual labeling or confirmation.

### Model Training

We use a computer vision model to improve the efficiency of our labeling process. Previous work has shown that such models can be trained to effectively identify and analyze CAFO locations^19,20,23,24^. Unlike other works, however, we supplement these detections with multiple rounds of human labeling for confirmation of buildings as CAFOs. We use the model to significantly reduce our labeling burden by separating and stratifying the population of images to label according to the likelihood of containing a CAFO. This hybrid approach provides a much stronger claim to a population enumeration of CAFOs and estimates of underpermitting than prior studies.

#### Training Dataset Collection

We train our model on a set of known CAFO locations from several states. The sources of these CAFO locations are satellite image surveys of Midwestern states by the Environmental Working Group (Indiana, Ohio, Michigan, Wisconsin)^25^ and the Socially Responsible Agriculture Project (Illinois)^26^. We also incorporate permitted CAFO locations from Wisconsin^27^, Iowa^28^ and California^18^, states with large numbers of cattle CAFOs. While Midwestern CAFOs and California CAFOs with accurate permit data may not necessarily be perfect representatives of the total CAFO population of California, they are the closest available visual proxies, and we sought to expose our models to a wide variety of facility appearances in hopes of increasing the recall of higher-confidence model detections for downstream confirmation and annotation by human labelers.

We provided 1 km x 1 km tiles of 1 meter per pixel satellite imagery from the National Agriculture Imagery Program (NAIP)^17^ centered on these known CAFO locations to Amazon Mechanical Turk workers, with instructions to place bounding boxes around the buildings where animals are kept (see the Supplementary Information for the complete labeling instructions). Determining whether a given building houses animals may not always be obvious from satellite imagery, so these instructions included a link to the coordinate at the center of the image on Google Maps to allow them to access higher resolution satellite and street views as well as a set of heuristics illustrated in Fig. 3 to facilitate the annotation process. We use the resulting bounding boxes to train and evaluate our model. Our labeled training dataset includes 8,479 total CAFO facilities, 12,521 images, and 57,236 building bounding boxes.

#### Model Architecture and Training

We used the YOLOv3 object detection implementation from Ultralytics as our base model^29,30^. This model takes its architecture from the You Only Look Once network^31^ and is pre-trained on ImageNet^29^. The YOLOv3 model was chosen for three qualities that were crucial to our analysis. First, the model was one of the most efficient and accurate object detection models at the time of training and inference (Fall of 2020): larger, more advanced segmentation models like Segment Anything and the geospatial adaptation, SAM-GEO were not released at the time of model training for this project. Second, the Ultralytics implementation is a fully-featured and open-sourced codebase. And, third, our computational resources were limited at the time, requiring us to choose a model that would be cost-effective to fine-tune. We then fine-tuned this model on our CAFO building bounding box dataset. The supplementary materials contains more detailed information about the parameters used for training our YOLO model.

### Detection and Labeling

In order to develop our statewide CAFO dataset, detections from the fine-tuned object detection model were used to efficiently prioritize more extensive human review efforts of satellite imagery covering the entire state. These efforts involved several layers of human labeling and annotation, each augmenting and further verifying the CAFOs we report.

#### Detection

We collected NAIP images covering all of California for the years 2016–2018 and created ≈1 km^2^ tiles. To allow for stratification by county characteristics, tiles along county boundaries were duplicated, with one entry for each county, and a mask limiting each image to its respective county. We masked Census-designated urban areas^32^ (using geographies from the 2010 census) from the imagery and removed tiles that were above 70% masked. This created a final pool of 496,093 image tiles for inference. We then applied our trained model to the urban-masked NAIP imagery of California to detect CAFO buildings, yielding bounding boxes and uncalibrated model confidence scores for all model detections.

##### Urban masking

Urban areas can be a source of false positives due to model detections of warehouses and other urban structures that resemble CAFOs. To reduce labeling burden, we removed the 2010 Census-designated urban area geographies, which cover 5.08% of the state’s area. To further validate this assumption, we applied our complete process (from inference to human annotation) to a number of urban areas, including those where urban/rural boundaries have shifted (like Chino, CA) and areas where sparsely populated suburbs intermingle with agriculture (like Petaluma, CA). A small percentage of the facilities we report (0.66%) are from within these urban boundaries. Conservatively assuming the same positivity rate for unlabeled and labeled parts of our urban mask, we would expect just 2 additional facilities. We do not include images in any urban masked areas for the purposes of our population estimation, but we include the small number of facilities found within urban masks in our dataset for completeness.

##### Other Common False Positives

Rural buildings that have similar characteristics to animal barns created a number of false positives which our human annotation procedure removed. For example, greenhouse structures are similarly large, long buildings but do not have key features like ventilation or feed storage. These buildings were removed by the human verifiers. Other types of animal storage buildings were detected by the model but do not constitute CAFOs due to animal type (for example horse stables) and/or facility size. These false positives were labeled by the human team as AFOs (animal feeding operations), not CAFOs, and are removed from the final dataset. The full dataset including these rural false positives is available at request.

#### Verification of CAFO locations

An initial layer of human review was used to confirm and refine CAFO locations based on our model detections as well as locations directly associated with CAFO permits. This step involved a comprehensive verification of all 20,599 images meeting one of three criteria: First, tiles containing known permit coordinates; second, those containing at least one high-confidence model detection (specifically, those with a confidence score of at least 0.5); and third – because facilities are often split across multiple images and geographically clustered – we additionally manually reviewed every image that was geographically adjacent to any CAFOs confirmed in the first two categories. This final step of labeling images adjacent to known facilities helped us capture a greater number of complete facilities and ensures we capture the full extent of facilities we find. Note that this verification process did not involve a mere sample of images that meet these criteria; we labeled the entire population of such images.

The selected images were shown to a human at a third party data labeling service, CloudFactory, chosen in part because of their ability to train and supervise a team on the specific task. The reviewers were instructed to confirm whether the image has a CAFO and if so, label each of its buildings with bounding boxes, using the decision flowchart in Fig. 3 and other resources. If the labelers were unable to confirm a suspected facility was a CAFO based on the NAIP imagery, we instructed them to first look at the location in Google Maps satellite view (which is higher resolution than NAIP imagery) for visual indicators, including facility labels. If that did not clarify it, the location (coordinates or address) was entered into a Google search and/or ReGrid to find indications of land use and/or potential owner information. Then, once a facility was confirmed to be a CAFO, labelers determined which building(s) house animals again using Google Maps (https://www.google.com/maps) satellite imagery and Google Streetview (https://www.google.com/streetview/) for a street-level perspective. The Cohen’s kappa for inter-rater reliability across the universe of sampled data for only the initial phase of labeling was 0.73. The kappa score only measures the reliability of one stage of our process, the initial labeling. Each CAFO in our dataset was verified in three separate stages; once during initial labeling, once during construction dating, and once again when animal typing. The high kappa combined with multiple layers of review demonstrates the reliability of our dataset. Our full process for measuring the inter-rater reliability is described in the supplementary materials Matching the labeled positives with facility permits and then further human review (see Section Permit, Animal Type, and Construction Annotation) gives us strong confidence in the stated completeness of our inventory.

Our resulting dataset contains detailed information on individual building locations and geometries within a given CAFO facility. By contrast, most existing data sources provide CAFO point locations, which may represent a street address, location of an administrative office, or a facility centroid. Our thorough annotation procedure ensures that the geographic information provided in our dataset reflects where animals are actually located, facilitating the investigation of research questions that depend on spatial relationships, facility footprint, and building orientation.

#### Population sampling to assess completeness

The procedure above was designed to efficiently annotate as nearly a complete accounting of California CAFOs as possible. In this initial step, we exhaustively label just 4.30% of the imagery covering rural areas in the state. This labeling effort captures 99.737% of the positive images reported in our dataset.

We ultimately found that our initial labeling effort was highly complete, but we knew that it might miss a small number of unpermitted, undetected facilities. In order to assess the completeness of our labeling effort, we constructed a population estimate of the total number of CAFOs in unlabeled rural areas in the state. To do this, we drew a stratified sample of the remaining population of image tiles. First, we stratified these images into two categories based on the presence of model detections:

1. **low confidence detection**, contains at least one model detection with an uncalibrated confidence score <0.5.
2. **no detection**, contains no model detections at all.

We further stratify these categories into thirteen county clusters. Each county cluster is made up of the images from a different set of counties, loosely clustered by region, yielding a total of twenty-six differently sized strata. We set initial sample rates from the strata relative to a prior based on the presence of known CAFOs in the geography. We then iteratively re-sample without replacement from the strata based on the relative prevalence of positive labels among strata, sampling randomly each time. These labels are then clustered, filtered and re-labeled according to the procedures in the following section so they may not appear in our dataset but the initial labels guided our sample procedure to ensure we maximized the completeness estimate of our dataset based on the labeling of the strata. See Table S1 for a final breakdown of the bins that images are sorted into, the resulting sampling rates for each and the final positives in each strata. In total, we sample 26,925 1 km^2^ images, which were annotated by the third party data labeling service following the same procedure described for verifying CAFO detections above.

#### Detection clustering

Prior mapping efforts have generally focused on building-level information^20^ or location-level information^19,24^. Developing a facility-level view of these locations allows researchers to perform analysis at the level relevant for regulatory and policy purposes. Collating building-level annotations into facility data, and splitting annotations into facilities independent of their permitting status additionally allows linkage between facilities, owners, and permits, which is not possible with unstructured building footprint or location data alone.

In order to develop such a facility-level aggregation of our CA CAFO dataset, we define a *facility* as a group of one or more buildings which are operated together as an animal feeding operation. Physical proximity and common ownership are both necessary conditions for a group of buildings to be considered a single animal feeding facility. This is consistent with the California Central Valley Water Board’s concept of “essentially one operation”; “contiguous border and/or common ownership” are indicators of a facility which is one operation^33^. We use two different pieces of information to distinguish adjacent facilities from each other: building bounding box distance and parcel ownership data.

Because parcel data was obtained from a third-party vendor (Regrid) at different time points during the development of our dataset, the date of parcel data access is reported when possible. When facilities straddle parcel boundaries, we report this information for each associated parcel. We also report the facility’s Census tract to facilitate linkage with other sources of data. We count a facility as ‘in’ the Census tract and county of a plurality of its building centroids. There were no ties at the tract and county level. There were nine ties at the census block level, which we broke randomly. Some buildings did not match to Regrid parcel data, so 131 facilities may have incomplete parcel information.

Based on our review of facility imagery and known permit locations, we developed a set of heuristics to determine whether any two buildings belong to the same facility:

- The buildings are on the same parcel
- The buildings are within 400 m of each other, and their parcel owner names are similar based on string-matching metrics
- The buildings are within 200 m of each other, and one of the buildings lacks parcel ownership data
- The buildings are within 50 m of each other, and one of the buildings is a ‘lone’ building (it is not related to any other buildings on the three criteria above)

The supplementary materials provides more detail on each of these criteria, including the choice of numerical thresholds and sensitivity to their specific values.

### Permit, Animal Type, and Construction Annotation

The specific risks CAFOs pose to the environment and public health vary by the type of animal housed^2,34^. Researchers can better measure these impacts by understanding how CAFO locations vary not just over space but also over time (enabling longitudinal and panel inference). We augment our facility list with additional data about each facility’s regulatory status, animal type, and changes over time. The sections below provide details about our methods for enriching our dataset with this information.

We record information about facility use based on heuristics about its visual appearance and permit status. These additional rounds of human labeling of detected facilities also provided an additional degree of validation of our dataset. A facility is only included in our dataset if three different indicators (the initial labeler who created building geometries, either a human labeler or a linkage to permit data to provide animal type information, and the construction dating labeler) all agreed that a facility was a CAFO. Because of the complexity of the process as a whole, note that we focused our measurement of inter-rater reliability on the first phase, the initial labeler. By reducing reliance on the judgment of any one human, the overall process would likely have even higher consistency.

#### Permit Matching

Publicly available permit data were obtained from the California State Water Resources Control Board^18^ (SWRCB) and matched to our facility list based on geographic information and parcel records. Matching detected facilities to permit data is not straightforward; in a random sample of 80 California Water Board permits, we found that the latitude and longitude registered on a permit only matched directly to a facility 30% of the time. Because the permit location data are inconsistent, we develop two procedures for reporting relationships between facilities in our dataset and administrative records: high-quality *best* permit matches, and more inclusive *expanded* permit matches.

Additionally, although the SWRCB permit data includes an indication of whether a regulatory program under which the permit was issued is currently active or inactive, our review of facilities suggested that inactive regulatory programs may still have currently operative facilities grandfathered into being approved. As such, we include matches regardless of this regulatory program status indicator and consider a facility to be permitted if it can be linked to any of these records.

##### Best permit matches

Best permit matches are permits that appear to be unambiguously related to one facility. A facility may have multiple permits (older permits, or distinct permits for two different parts of a facility, for example), but a permit can only be associated with one facility. We consider a permit to unambiguously match with a facility if both the location of the registered permit and the address given by the permit (geocoded with Google Maps) are on the same parcel or within 200m of a facility, and are not both within 200m of any other facility. Using this criteria, 1,935 permits match to a facility. The supplementary materials describe the process we used to establish this threshold.

##### Expanded permit matches

Expanded permit matches are intended to be a complete and inclusive list of any permit that could be associated with a facility; any permit whose registered location or address is within 1,000 meters of a facility is included in the expanded permit match list. Unlike ‘best’ permit matches, one permit may be included in the ‘expanded permit match’ list for more than one facility.

#### Animal Typing

We use information from California’s CAFO wastewater permits and human labeling to determine the type(s) of animals each facility houses. California AFO permits classify whether each facility contains cattle and/or “other” animals^33^. If the facility has a high-confidence match to a cattle permit, we assume that it is a cattle facility. If not, a human inspects the facility from aerial imagery and any information available on Google Maps, and uses this information to identify the kind of animals that it houses (‘cattle’, ‘dairy’, ‘poultry’, ‘hog’, ‘other’, etc.). As described in Table 2, it is not always possible to affirmatively animal type every facility. When we are unable to do so, an ‘unknown’ animal type label is included, indicating uncertainty about the animal type. Finally, we include an indicator which states whether the animal type label was derived from a permit or from human annotation.

**Table 2:** Building on previous animal facility classification work in other geographies^40^, we captured several heuristics for classifying California facilities by type (CAFO, AFO) and variety of animal contained.

Required Resolution	Features	Facility Type	Animal Type
Low Res.	NOT LIKELY AN ANIMAL FEEDING FACILITY		
	Parking Lot	none	n/a
	Shooting Ranges	none	n/a
	Grain Silo	none	n/a
	LIKELY A SMALL ANIMAL FEEDING FACILITY		
	Training Rings	AFO	horse
	Racetracks	AFO	horse
	BUILDING CHARACTERISTICS		
	Narrow Shed	(C)AFO	poultry
	Wide Shed	(C)AFO	cattle/swine
	Open-sided Shed	(C)AFO	cattle/swine
	FEED STORAGE		
	Dispersed Covered Feed	CAFO	cattle/swine
	Rolled Covered Feed	(C)AFO	cattle/swine
	WASTE DISPOSAL		
	Lagoon	CAFO	cattle/swine
Medium Res.	NOT LIKELY AN ANIMAL FEEDING FACILITY		
	Nursery	none	n/a
	CONTAINMENT AREA		
	Trampled Earth	(C)AFO	cattle/swine
	Tracks	(C)AFO	cattle/swine
	Outdoor Pens	(C)AFO	cattle/swine
	FEED STORAGE		
	Feeder Silo	CAFO	inconclusive
	Feed Ring	AFO	cattle/swine
	Feeding Troughs	(C)AFO	cattle
	Dry Litter Storage	(C)AFO	poultry
High Res.	Animals	(C)AFO	cattle/swine
	Shed Ventilation	CAFO	any
	Underground Lagoon Ports	CAFO	swine

All facilities need to contain, feed and deal with animal waste. At each resolution, we list indicators of non-animal facilities (features that can be confused with animal facility features, e.g. plant nurseries, or features that rarely co-occur with animals, e.g. large parking lots), as well as features indicative of how the animals are contained, how their food is stored, and how the animal waste is managed. Because each characteristic is indicative, but not conclusive on its own, if we are unable to visually confirm *a few* features, the facility is classified as ‘unknown’. More detailed information including sample imagery can be made available upon request.

In our dataset, we label the 459 facilities that have one or more best permit matches to only cattle permits as cattle facilities. For the remaining 1,662 facilities that either do not have a best permit match or match to any “other” animal permit, we manually labeled their animal types using a set of heuristics listed in Table 2. Manual labelers used visual features of the facility from high-resolution Google Earth satellite imagery, Google StreetView imagery, and Google Maps location labels to identify animal type.

##### Distinguishing cattle and dairy facilities

Our animal type labeling has two categories, ‘cattle’ and ‘dairy’, that are not mutually exclusive. Dairy facilities are a subset of cattle facilities. All facilities marked both ‘cattle’ and ‘dairy’ are certainly dairy facilities; these labels come from human annotators and include heuristics such as the Google Maps description or name of the facility. Facilities marked ‘cattle’ alone may be either beef cattle or dairy facilities. It is not always possible to unambiguously determine the nature of the operation from satellite imagery, and we do not attempt to apply our animal typing heuristics to facilities with cattle permits. However, labelers also collected information about whether animals are housed indoors or outdoors, which can be a helpful indicator of facility type; dairy cattle are more likely to be housed in enclosed barns. Compare, for example, APHIS’ 2007 study of dairy facilities^35^ (page 87) and the USDA’s description of cattle in the Southwest^36^.

#### Facility Use and Features

Each facility is annotated with an operational date range (the estimated bounds of the facility’s construction and destruction dates) based on presence of animals and buildings in available aerial imagery (Google Earth Pro’s historical imagery: https://bit.ly/4p06q23), and whether any significant change in population occurred during the operational period. Reflecting the intermittent collection of remote sensing imagery (particularly for earlier time periods), this operational range is defined by four dates: a construction lower bound, a construction upper bound, a destruction lower bound, and a destruction upper bound.

**Fig. 4:**
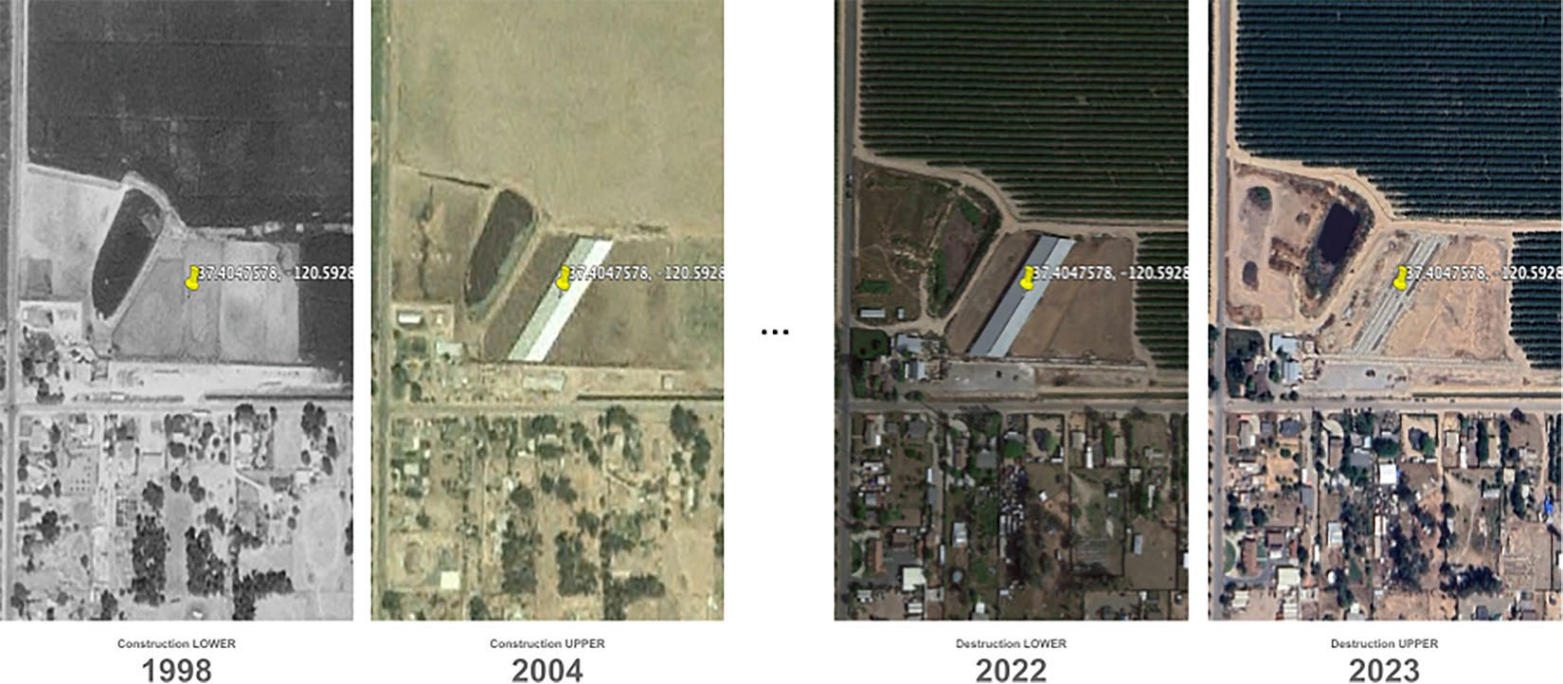
An example of construction dating for a facility in California, showing the relationship between animal presence in satellite imagery and construction lower/upper and destruction lower/upper bounds in the dataset. The left panels depict the construction of a facility between 1998–2004 and the right panels depict the destruction of a facility between 2022–2023.

**Fig. 5:**
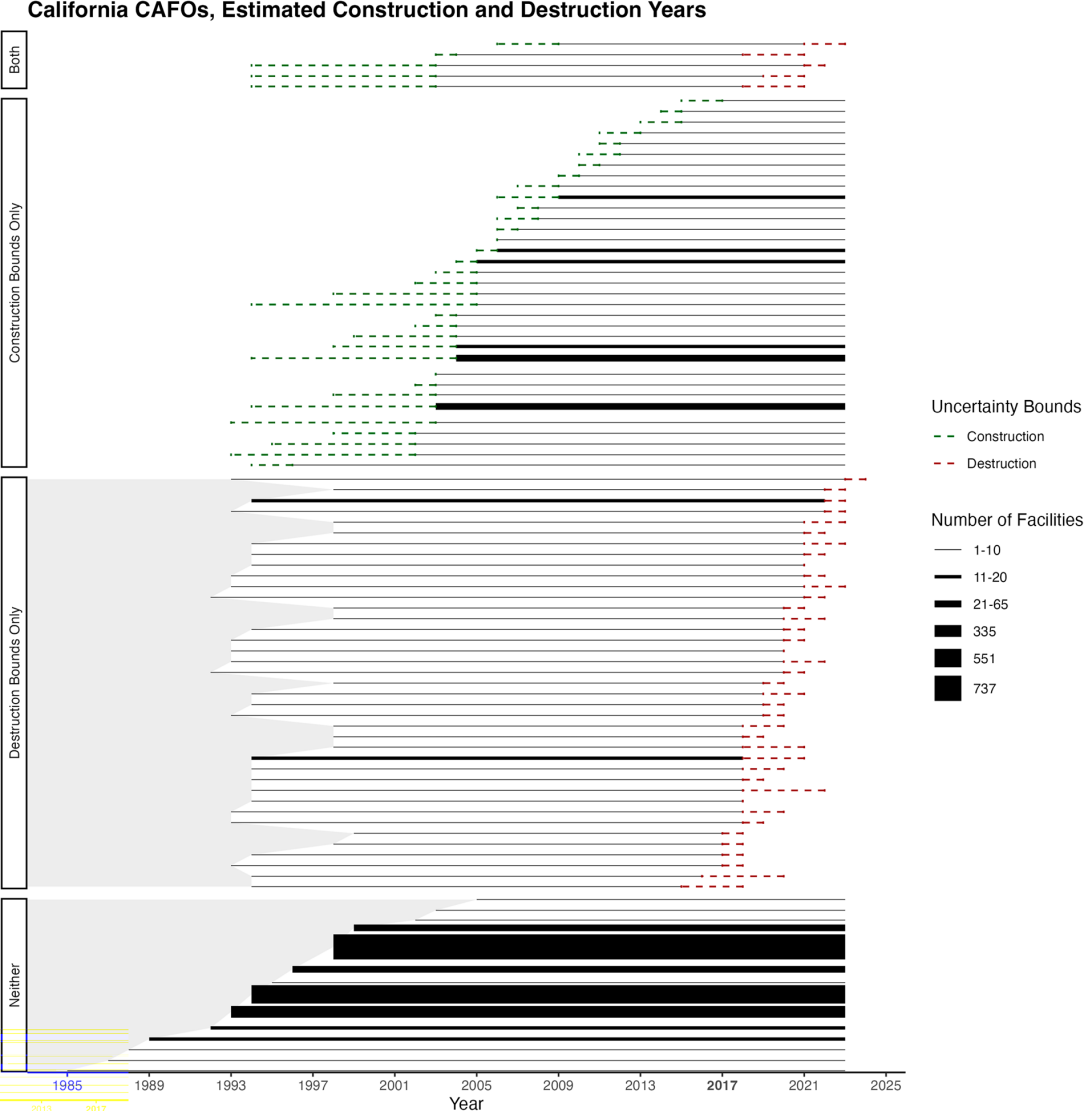
Estimated construction and destruction dates for the n = 2,121 CAFOs detected in California. CAFOs are detected in 2017 imagery; imagery is then traced forward and backward in time to estimate construction and destruction dates. Each panel represents facilities that have similar construction and destruction profiles: “Construction Bounds Only” indicates the facility was built after the first available imagery and exists through the last available imagery (2022); “Destruction Bounds Only” contains facilities that existed before the first available imagery and ceased to exist at some point during available imagery; “Neither” captures facilities that existed before first available imagery and in last available imagery; “Both” has facilities that were built after our first available imagery and no longer exist at some point in available imagery. Gray shading to the left indicates time periods when facilities may have existed, but we do not have available imagery to check this. Within each panel, CAFOs with the same construction / destruction profiles are grouped into a single line, with line weight indicating the total number of CAFOs in that grouping. Green dashed lines represent the time range between the lower and upper bounds on the construction date based on available imagery; red dashed lines represent the time range between lower and upper bounds on the destruction date. Time between the upper bound of construction and lower bound of destruction (if relevant) is represented by a solid line, indicating the period of time in which we are most confident that the facility existed. For CAFOs with no evident destruction event, the terminus of the solid line has no dashed red bounds.The largest grouping of CAFOs is those that have a construction upper bound in 1998, with no construction lower bound (i.e. detected in all available imagery) and no evidence of destruction.

Figure 4 shows an example of a facility with construction (left) and destruction (right) events annotated. Because the labelers only captured the year of the imagery, we can safely assume that the first day of the year (at least) is one where the facility was not present. The construction upper bound is the earliest image that depicts the area with animals housed; all facilities have a valid construction upper bound. In other words, the construction upper bound is the first year where the facility is *known* to exist at a location; however, it should be noted that due to limited availability of satellite imagery in earlier years, this upper bound may be later than the true construction date. Similar to the lower bound, the full date given is the last day of the year. Because the labelers only wrote the year, we can safely assume that the last day (at least) is one where the facility was present. The destruction lower bound is the most recent image that depicts the area with animals housed. The destruction upper bound is the earliest image that depicts the area without animals housed. Facilities that do not experience evident destruction – i.e. are present through the latest imagery evaluated (2022) – have null values for both destruction lower and upper bound. In the dataset, the date given is the first day of the year. In the majority of cases, the facility has existed for the entire time imagery is available, and so have existed at least the mid 1990’s (with earliest known dates varying based on imagery coverage) (Fig. 5). We observe 162 destruction events and 208 construction events. Date ranges for construction events (time between the upper and lower bound) are a bit wider for the earliest constructions, given reduced cadency of historical imagery.

Figure 5 depicts the estimated construction and destruction dates, including periods of uncertainty, for facilities in the dataset. Note that for visualization purposes, one facility that is present in the dataset has been omitted from this figure because it has construction bounds far earlier than any other observations in the dataset; this facility has a construction lower bound in 1931 and construction upper bound in 1949.

As part of our human review effort, annotators were also instructed to indicate whether the facility is a feedlot – an almost entirely and open-air facility, whether animal population changed significantly between the construction and destruction dates, whether the animals are primarily housed indoors or outdoors, and whether the facility has a ‘lagoon’ (that is, an exposed outdoor manure disposal pool).

## Data Records

The dataset is available on the Hugging Face data repository^37^. Each record in our dataset represents one CAFO facility in California. For each facility, we report information about (1) geography, (2) active usage periods, (3) type of animals contained, (4) land information, and (5) regulatory status. Our primary data release is in two formats: a flat CSV intended for convenient access to key features, and a rich GeoJSON file (an open standard for storing geographic information) which contains all information. We also release source data files and code for reproducibility and additional flexibility.

### facilities.csv

The facility CSV file is intended to be a convenient representation of high-level information about each facility in our dataset. It is a flat file, meaning that it has no nested information or information about one-to-many relationships. This CSV contains the following fields for 2,121 records: a unique facility ID, location of the facility centroid (latitude, longitude), bounding box of all associated buildings (lat_min, lon_min, lat_max, lon_max), n_buildings, footprint_sq_m (the area of all buildings), county, zip_code, census_tract, census_blockgroup, animal_type, construction_lower, construction_upper, destruction_lower, and destruction_upper. Facilities that straddle county, zip, or census tract boundaries are associated with the division that contains a plurality of building centroids.

### facilities.geojson

**Fig. 6:**
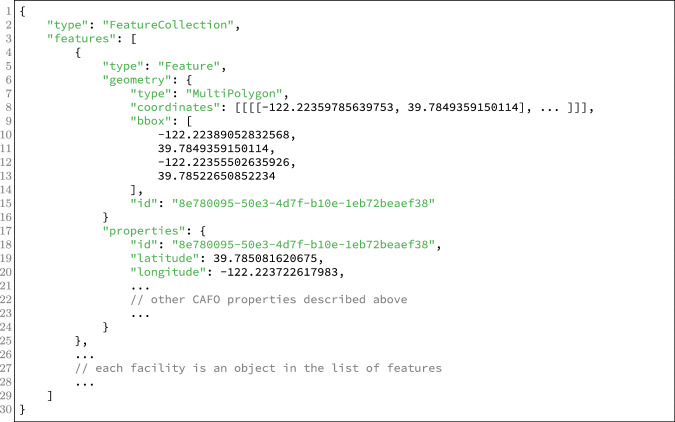
An example of the structure and content of the GeoJSON file, our rich data format.

This file is intended to represent all available information about a facility, including detailed geometries and one-to-many relationships. It is structured as a GeoJSON feature collection. Import and export are supported by many libraries and applications, including ArcGIS. An example of our GeoJSON output is shown in Fig. 6.

In addition to each field present in facilities.csv, our repository also contains:

- A GeoJSON MultiPolygon feature representing the complete building footprint information for each facility; each building is a polygon of the MultiPolygon.
- A complete JSON list of parcels associated with each building in the facility, each with number, county, and zip_code.
- A JSON list of best permit matches, each with wdid (California’s permittee identifier), regulatory_program, activation_date, termination_date, facility_name, facility_address, agency_name, agency_address, npdes_no, cafo_population, latitude, and longitude, as provided by state records.
- A JSON list of expanded permit matches, with the same fields.
- A JSON object census_block with countyfp, tractce, blockce and geoid

### Data Features

The primary geographic feature in each GeoJSON record is a MultiPolygon which represents the footprint of each building in the facility. For convenience, we also calculate several statistics about each facility’s geographic extent and include them in both the .csv and .geojson files. These statistics include the centroid of the facility (determined by the centroid of the MultiPolygon representing all buildings: properties → latitude, longitude), the axis-aligned bounding box of the facility’s buildings (properties → lat_min, lat_max, lon_min, lon_max), and a facility’s total building footprint can quickly be calculated from the MultiPolygon coordinates (or the footprint_sq_mi field in the csv).

#### Land information

We report information about the parcel(s) underlying each facility under the properties → parcels heading. These public data include the parcel county and number. This data was collected from the Regrid Property app (https://app.regrid.com/) by submitting the centroids of the labeled buildings to Regrid and retrieving the csv of the parcels that the buildings reside on. To protect privacy, we withhold the names of parcel owners in our public release.

#### Regulatory Status

The fields within properties → best_permit_matches, expanded_permit_matches (defined in Sec. Permit Matching) are additional features from the Clean Water Act permits issued by the California SWRCB that can be associated with each facility. There is a one-to-many relationship between facilities and best permit matches. Each permit can be the ‘best match’ of only one facility, and appear in only one facility row. A given facility may be a ‘best match’ for multiple permits. There is a many-to-many relationship between facilities and expanded permit matches. Each permit can belong in multiple ‘expanded permit’ lists.

## Technical Validation

We verify that our dataset is both accurate (meaning that each reported facility is truly a large animal feeding operation) and complete (meaning that the set of reported facilities captures all or nearly all facilities that exist in the state of California) by labeling each entry multiple times and robustly estimating labeling completeness.

### Manual review

One of the key improvements of our dataset over prior model-prediction datasets is the rigorous human labeling we perform to ensure that each facility we report is a CAFO per our definition requiring visual evidence of large-scale animal housing. We confirm that every facility in our dataset is a CAFO in at least three ways. First, each building is annotated by a human in the labeling stage. Annotators are instructed to only label buildings which have indicators of housing large numbers of animals. The initial labeling process had a Cohen’s kappa score (a measure of inter-rater reliability) of 0.73. This is consistent with our expectation that the labeling task is well-defined, with some subjectivity around the boundary between large AFOs and small CAFOs. All annotations were reviewed by at least one other person before being advanced to the next step.

In total, we labeled 47,523 satellite images, representing around 9.97% of the rural area of California, including our initial high-confidence labeling as well as our our wide sampling to build confidence in completeness. Among these images we identified 37,518 buildings as potential CAFO animal holding buildings, which our clustering algorithm aggregated into 2,721 facilities. Each facility was then sent to annotators for construction dating. During the construction dating process, annotators also had the ability to indicate that a facility lacks the visual indicators of large-scale animal housing. Reviewers at this stage disagreed with the initial assessment for only 2.28% of initial facilities. This results in the removal of 62 facilities in the final dataset. These changes were largely due to disagreement about whether an AFO was large enough to meet our definition of CAFO for the purposes of this dataset. Our dataset only includes facilities which all annotators agreed were large enough to be a CAFO. These annotations serve as a second confirmation that a facility is a CAFO per our definition.

The animal typing process serves as the third check. Facilities are confirmed in one of two ways: each facility is either matched with high confidence to a wastewater permit, or it is manually labeled by a human. When a human labels the animal type, they are able to indicate that the facility does not have the indicators of a CAFO. This final check removed 506 (18.60%) of facilities initially labeled as CAFOs because they did not appear to house animals at sufficient levels to qualify as a CAFO.

Together, these three checks made use of a variety of data sources (administrative records, satellite imagery, and ground-level street view images) and multiple human labelers to enrich the precision of our dataset, increasing our confidence that each facility we report is in fact a substantial animal agriculture operation.

Among the 2,121 total facility clusters detected in the imagery, 1,166 (55.0%) have a good match to a permit, 955 (45.0%) do not have any higher-confidence “best” permit matches, and 157 (7.4%) are not within 1km of any permit (and therefore appear to be potentially unpermitted operations).

### Labeling completeness

The manual validation processes described above focused on reducing false positives in our dataset, but it is also possible that our procedure for efficiently surfacing candidate facilities for review could yield some false negatives as well. In order to understand the completeness of our dataset, we develop a protocol to estimate recall. To do so, we employ a geographic and model-confidence based stratified sampling and population estimation procedure for low-confidence and no-detection images, drawing images from a total of 26 strata as described in the Methods section. Table S1 describes the total number of images in each stratum, the number of images we labeled and the number of positives found in the stratum after all labeling was finished.

We estimate the total population by separately estimating the proportion of positive images in all the low-confidence strata and no-detection strata and multiplying this estimated proportion by the total number of unlabeled images in each category of strata. We sum these two estimates together to arrive at a total estimate for the unseen population, which we add to the known, observed population to estimate the population in the state overall.

#### Proportion estimates

Because the prevalence of facilities is very low in all strata and exactly 0 in many, estimating variance with the binomial distribution is inappropriate. We hence use the method proposed by Waller *et al*.^38^ to calculate a mean and 95% confidence interval estimate for the proportion of positive images in each stratum. The method takes a weighted sum of the confidence intervals for each stratum to create a total confidence interval for the whole population.

Using this method, the lower (*LB*) and upper (*UB*) bounds of the population proportion are given by:

1 $$LB=\widehat{p}\,-\,(\widehat{p}\,-\,L{B}_{0})R$$

2 $$UB=\widehat{p}+(U{B}_{0}\,-\,\widehat{p})R$$

with:

3 $$R=\frac{{({\sum }_{i}^{L}\frac{{w}_{i}^{2}}{{n}_{i}})}^{\frac{1}{2}}}{{\sum }_{i}^{L}\frac{{w}_{i}}{\sqrt{{n}_{i}}}}$$

4 $$L{B}_{0}=\mathop{\sum }\limits_{i=1}^{L}\frac{{w}_{i}{x}_{i}}{{x}_{i}+({n}_{i}-{x}_{i}+1)F(\frac{\alpha }{2},2({n}_{i}-{x}_{i}+1),2{x}_{i})}$$

and,

5 $$U{B}_{0}=\mathop{\sum }\limits_{i=1}^{L}\frac{{w}_{i}({x}_{i}+1)F(\frac{\alpha }{2},2({x}_{i}+1),2({n}_{i}-{x}_{i}))}{({n}_{i}-{x}_{i})+({x}_{i}+1)F(\frac{\alpha }{2},2({x}_{i}+1),2({n}_{i}-{x}_{i}))}$$

where:

*L* is the number of strata,

*w*_*i*_ is the weight associated with the *i*-th stratum, usually the proportion of total elements in the strata,

*x*_*i*_ is the number of positive labels in the *i*-th stratum

*n*_*i*_ is the total number of labeled elements in the *i*-th stratum

$\widehat{p}$ is an unbiased estimator for the population proportion; in this case, the weighted sum of the proportion observed in each stratum

*α* is the significance level, in our case, 0.05

*F*( ⋅ , ⋅ , ⋅ ) is the F-distribution percent point function

Because Waller’s method assumes the population variance is similar across strata, we apply the equations above *separately* for all strata with no model detections and all strata with low confidence detections. Doing so yields upper and lower bounds on the proportion in each group, which are then summed together to yield estimated bounds on the total number of facilities not identified by our procedure.

For the no-detection strata, this yields a point estimate for the proportion of positive images of 0.0001, with an upper bound of 0.0013. With 393,355 unlabeled images in this strata, we estimate the upper bound of unobserved positive images to be round(0.0013 × 393, 355) = 517. Applying the same procedure to the low-confidence detection strata, we arrive at a population estimate of 36 unobserved positive images, with an upper bound of 148 unobserved positives. Together with the images labeled through our protocol, this gives us an estimate of the total number of tiled NAIP images that contain a CAFO: 3,129 with upper bound of 3,716 images.

#### Facility estimates

The uncertainty bounds above estimate the completeness of our labeling of image *tiles*. However, an individual facility may span multiple tiles or a given tile may capture multiple nearby facilities. We hence convert these image-level population estimates into an upper and lower bound on the number of CAFOs in California. Among the set of labeled, positive image tiles, there is a ratio of 1 facility to 1.44 images. This statement is not quite the same as ‘the average facility is spread across 1.44 images,’ as images can have more than one facility on them. 10 facilities may lie on the same two images. The average facility in that case would occupy 2 images, but there would be 1 facility for every 0.2 images, since the images are so heavily ‘shared’ between facilities. While it may be that unobserved facilities look different from observed ones, we use this statistic as a heuristic to estimate the number of unobserved facilities in California. We assume that there is one facility for every 1.44 unobserved positive images. Applying this to our estimate of unobserved positive images, we estimate that there are 2,172 facilities in California, of which 2,121 are labeled and included in our dataset. Our point estimate for the completeness of our dataset is 98%; with 95% confidence interval [82%, 98%]. This very high rate of coverage reflects the advantages of our procedure in combining signals across administrative permit records, satellite imagery, and the geographic clustering of livestock operations to search for these facilities.

## Usage Notes

Our California CAFO dataset offers a unique opportunity to investigate questions related to environmental pollution, policy and regulation, community exposures, environmental justice, and public health. In addition, the underlying methodology for compiling these data can be applied to map CAFOs and other similar sources of environmental risks in different geographic contexts. Future studies exploring these research questions are likely best investigated by augmenting this California CAFO dataset with linkages to other data sources, such as air and water pollution concentration measurements, meteorology information, or human health and community demographic records. This work would also create a head start for similar efforts in other places. Though many of the administrative records we use are unique to California, our image detection and labeling protocol is generalizable, and our final dataset of human-validated bounding boxes would be an important resource for training new satellite imagery detection models.

We see the richness of the metadata we curated for this dataset as a particularly valuable asset for such studies. By providing detailed information on building locations and polygons, construction and destruction dates, and animal types, these data allow for new avenues of research not feasible with existing CAFO datasets, which focus only on enumerating facility locations at a fixed point in time. As compared to point locations or facility centroids, building locations and polygons allow greater insight into the relative extent and intensity of different facilities and may allow for more advanced exposure assessment. Our dataset is the first to report human-annotated construction and destruction dates for CAFOs, thereby enabling stronger inferences about the environmental and social impacts of CAFOs. This allows researchers to make comparisons using active CAFOs and pre/post CAFO construction, if applicable. Construction and destruction dates are necessary for establishing temporal relationships related to the presence or absence of facilities and may enable more robust approaches to inferring causal impacts of CAFOs. Finally, different animal types may be related to distinct exposure profiles, waste-disposal practices, and animal-rearing methods; the labels in our dataset allow for filtering and comparisons based on this feature.

Comparing our carefully curated dataset to available administrative records also highlights the potential confounding introduced into studies that rely solely on permits and administrative data. The SWRCB data we obtained contain a total of 3,761 unique permits. This includes 2,537 larger facilities with registered animal counts >200, 677 facilities with registered animal count <200, and 547 facilities with no registered animal count. This suggests a much larger population of facilities relative to the 2,121 we identify here. Yet upon examination, these records include substantial duplication, expired or outdated permits, and incorrect location information.

In our labeling process, we manually labeled all locations associated with these permits, regardless of size, including both the permit address and registered latitude/longitude. We found that 1,826 permits have no “best match” facility in our data, meaning that they did not match unambiguously to a single facility. On the more generous expanded matching criteria, 687 permits had no CAFO within 1km of their registered location or geocoded address. 134 of these 687 permits reported animal counts >200 and had no recorded termination date. At these permit locations, we found an assortment of residential subdivisions (which may have recently been developed where a CAFO previously stood), empty fields, and vineyards.

In addition to the identification of additional, unpermitted CAFOs, the manual verification of permitted locations is valuable, as geospatial analyses relying on noisy records could be substantially misled.

### Limitations

We note several limitations. First, facility clusters have a degree of uncertainty. Distinct facilities on adjacent parcels may be incorrectly aggregated together while operations with a large spatial extent or spread-out buildings could be clustered as separate CAFOs. We sought to tune the parameters of our facility clustering algorithm to as accurately as possible group buildings with common owners and operators (see the Supplemental Methods for details), but different data applications may require more or less permissive grouping. Our full data release includes information on individual buildings and the relationships that we noted between buildings, and researchers may choose to manipulate these parameters to suit their needs.

Second, we relied on the 2017 NAIP imagery, so facilities constructed after 2017 or closed prior to that date may not be captured in the data. In areas where residential growth has replaced CAFO facilities, the 2017 imagery may not fully capture historical CAFO activity in that area. For instance, near the city of Chino in San Bernardino County, residential subdivisions replaced a historically large number of CAFOs (and many Water Board permits in that area do not match current facilities). That said, a majority of facilities detected in 2017 exist for the entire duration of time that satellite imagery was available, which in most cases is a period of more than 20 years, from the 1990s through 2020s (1,757 facilities, or 82.8% of all facilities detected, have neither construction nor destruction bounds, and therefore exist for the entire duration of available imagery) (Fig. 5). Thus, these data can be used in many varied study designs, including those with spatial and temporal aspects. Future work could extend our approach to both earlier and later waves of remote sensing data to supplement this dataset with a more complete temporal view of the CAFO population.

Finally, while we provide information from permits about animal units, such estimates are known to fluctuate at least seasonally due to varying maturation times for individual species. Further, California CAFO operations present distinct challenges: In many states, animals are primarily housed indoors, meaning building footprints may correlate well with animal counts. In contrast, California’s relatively mild climate allows many facilities to rely more on outdoor feedlots, where the relationship between square footage and number of animals is likely more variable (in these cases, the facility buildings we annotate are often shade structures that do not directly house the animal population)^39^.

Exploring methods and data sources that can overcome this challenge is a fruitful direction for future work. Augmenting future data with information on facility size, intensity of production, and seasonality of animal rearing could aid efforts to quantify pollutant emissions and effects on neighboring communities. Further temporal analysis with this dataset could be enabled by training newer models on our human-validated bounding boxes and inferring new facility locations by running the updated pipeline with an improved CV model on newer NAIP imagery from California.

## Supplementary information

Supplementary Information

## Data Availability

Our dataset and raw input data is available on Hugging Face at https://huggingface.co/datasets/reglab/cal-ff(10.57967/hf/5118).

## Code availability

All code used to generate the data and figures in this paper is available at https://github.com/reglab/cal-ff.
