# Supplementary material for: A Comprehensive Dataset of Factory Farms in California Compiled Using Computer Vision and Human Validation
Source: Sci Data. 2025 Nov 19;12:1826. doi: 10.1038/s41597-025-06082-6 (PMC12630652; doi:10.1038/s41597-025-06082-6)
Supplement: Supplementary file 1 — Supplementary Information [file 41597_2025_6082_MOESM1_ESM.pdf]

Supplementary Materials for "A Comprehensive Dataset of Factory Farms in California Compiled Using Computer Vision and Human Validation" (Magesh et al.)

Labeling Instructions for Model Training Data

As described in the Methods section (see “Training Dataset Collection”), we trained our model on known CAFO locations by providing imagery on these locations to Amazon Mechanical Turk workers. Here we include the full set of instructions given to the workers for the purposes of labeling and placing bounding boxes:

Instructions Select the appropriate facility size (CAFO/AFO) and animal type (cattle/swine/poultry/unknown). Draw a box enclosing the facility, including all associated structures (including sheds, feed silos, covered feed, and lagoons).

Multiple labels per image are allowed, as well as no labels. For the latter, select "nothing to label" from the bottom right corner.

For difficult decisions, you may follow a link to google maps corresponding to the ID in the upper left corner, provided in the job spreadsheet. If still unable to make a determination, choose the "Flag" label to mark the image for further study.

Examples Figure S2**(a)** is an example of a single cattle CAFO. Select the "CAFO: cattle" box label and draw such that all associated structures (including sheds, feed silos, covered feed, and lagoons) are enclosed.

Slightly more complicated, in Figure S2**(b)**, we see another cattle CAFO, but this time there are two nearby facilities with silos and sheds. These smaller facilities are AFOs, label them accordingly.

Figure S2**(c)** is an example of a poultry CAFO. Note the long narrow building, small feed silos, and lack of liquid waste storage. Choose the appropriate "poultry" label and box as usual.

The last major category is swine. Figures S2**(d)** and S2**(e)** are two examples, the second being rather minimal. Note the wide sheds and either lagoon or external underground lagoon ports.

Mixed types are definitely possible. In Figure S2**(f)** we see a swine and cattle facility next to one another. Make sure to use the appropriate label box for each.

Lastly, Figures S2**(g)** and S2**(h)** are images with no CAFOs present. Simply click the "nothing to label" button.

Object Detection Model Training

To train the YOLOv3 object detection model[1] we used to prioritize our manual verification process, we performed an image-level proportional split of 0.7 train, 0.1 validation, 0.2 test, uniformly sampling among the training images described in the Methods section of the main text. Each time an image is sampled, it is augmented using a random rotation, translation, rescaling, shear, hue, saturation and value. Our model was trained using the Adam optimizer[2] and weight decay. Model selection was performed using a fitness function on the validation set which used a weighted sum of mean average precision (mAP) and recall of predictions with intersection-over-union (IOU) above 0.6 and model confidence above 0.1. Following the approach employed in the initial pre-training of YOLOv3 on imagenet data,[1] we weight mAP at 0.99 and recall at 0.01.

Building Clustering Critera

As described in the Methods section in the main text, many CAFO facilities will straddle multiple property parcels. As such, we use a combination of distance between verified CAFO buildings and parcel ownership information to aggregate our building-level detections into facilities. Because parcels operated as a single facility may have different owner names on record (such as different family members, trusts, or even just variations in spelling), we allow for inexact matching between these names. Here we provide additional details about the parameters of this matching process, sensitivity of the final set to their values, how we chose them.

Parcel data and direct matching Parcel number and ownership data was obtained from Regrid (`http://regrid.com`) via API queries on the location of each verified CAFO building. As an initial aggregation step, all buildings which are co-located on the same parcel were assumed to be part of a single facility and aggregated together.

Distance & ownership matching In California, many adjacent parcels that are clearly the same facility have slightly different recorded owners; one common pattern is adjacent parcels owned by a person and a trust seemingly in their name. For this reason, we compare parcel ownership by using two quantitative measures of string similarity: Levenshtein distance and tf-idf. We use the Levenshtein distance, a measure of string similarity based on the edit distance between two strings, to perform fuzzy-matching of owner names — for example, CAT and CAB are more similar than CAT and DOG, because the former pair does not need to be changed as much to match. We augment this with a tf-idf similarity threshold. Tf-idf is a phrase vectorization method that compares the frequency of a term in a given document (in this case, a parcel owner name) compared with the frequency of that term among all documents — for example, two parcel owner records 'HELEN CORMORANT' and 'ROB CORMORANT' will have a high tf-idf similarity score because of a unique shared name, but two parcel owner records that only share a word like 'TRUST' or 'DAIRY' will have a low tf-idf similarity score, since the words that match are very common in the data.

We consider parcels that have a high tf-idf score, a high fuzzy matching score, and physical proximity to be under common ownership or control. This is because the owners are likely the same or are closely aligned. In order to reduce the chance of incorrectly aggregating distinct facilities, we chose high thresholds for tf-idf and fuzzy matches (0.7 and 0.6 respectively). We set each threshold by randomly sampling 2,000 pairs of nearby parcels with distinct parcel names for manual review by a human annotator to determine whether they were likely owned by the same group or not. To ensure the reviewed pairs provided sufficient variation on each dimension, sampling was performed in two batches: the first batch of 1,000 was stratified by tf-idf score, such that there were 100 samples for each decile of tf-idf. The second batch of 1,000 was stratified by fuzzy matching score decile. To identify a threshold for each parameter, the annotator labeled in descending order of tf-idf score until finding a pair that should not be considered the under common ownership. We then rounded up to the nearest decile, and set that as the lower bound of tf-idf. We repeated the same procedure for fuzzy matching, but rounded down to the nearest decile. We chose to be more permissive with the fuzzy matching because it will be AND-ed together with the more restrictive tf-idf criteria. Finally, we compared building groups before and after applying these string-similarity thresholds and identified 43 hand corrections to consider two parcels under common ownership even when they did not meet this threshold (See Supplementary Table S2 for a complete list of these additional pairs).

Tables S3 and S4 show examples of parcel owner names and their string matching scores. To understand the sensitivity of our results to these parameter choices, we investigated how the number of facility groups varied with each string matching threshold value. As seen in the right two panels of Supplementary Figure S3, relatively few clusters are affected by this choice, with the number of distinct groups only increasing by about 25 (less than 1%) even when requiring exact matches or a tf-idf score of 1.0.

Distance matching We chose our distance parameters by examining the sensitivity of our facility count to the distance parameter, as reported in the left panel of Supplementary Fig. S3. We observe an 'elbow', or a significant change in the effect of the distance parameter, at around 200 meters, which we use as a threshold in the absence of parcel ownership information. When ownership information is available, the risk of over-grouping is reduced and we relax this parameter considerably to 400 meters, as the ownership matching procedure described above gives us greater confidence that two buildings are under common ownership even when they are further apart.

Lone building matching Finally, we observed some instances of buildings very near a parcel boundary that were improperly geocoded, especially when they abut an oddly-shaped parcel boundary created by a waterway or a railway (see Supplementary Figure S4 for an example). In order to handle these 'edge' cases, we included a final criterion of grouping together lone buildings less than 50m apart.

Once building relationships are determined, we group buildings into facilities by grouping all buildings that are connected to each other, an equivalent to the graph concept of a connected component.

Permit Matching Threshold

A facility is considered a 'best' match for a permit if:

1. The permit's registered location is on a facility's parcel or within 200m of a facility
2. The geocoded location of the permit's registered address is on a parcel or within 200m of the same facility

3. Neither location is also within 200m of another facility

We determined that neither permit location nor geocoded address alone were reliable indicators of facility location. We came to this conclusion by sampling 80 random permits from the set of permits whose listed facility location and geocoded address locations did not map to the same land parcel and determining which (if any) location information matched a facility. In many cases, only one or neither location matched to a facility: for only 10 of these permits did both locations both map to a location within the same facility, in 47 cases only one of the two matched to a CAFO facility and in 13 cases neither location was at a CAFO facility. During this process, if one pointed to a CAFO facility and the other did not, we assume this is a match to that facility. For the purposes of the published dataset, in order to retain high confidence in our animal typing, we chose to adopt the requirement that both locations must match to the same facility and no other, reducing the possibility that the permit could belong to a different facility.

To determine a distance threshold for these matches, we performed a sensitivity analysis of the relationship between this parameter and the number of high-confidence matches identified. As shown in Supplementary Figure S5, the number of matches initially increases with distance (as more permits are associated with the closest facility) then, at greater distances, the permits started falling within the range of other facilities as well, causing the number of high-confidence matches to fall off. This suggests a balance between allowing a sufficiently-large threshold to account for permit locations that may not directly correspond to CAFO buildings while not using such a large distance that permits are haphazardly associated with any relatively nearby facility. Based on this analysis, we chose a threshold of 200 meters, near the maximum of this relationship but slightly on the conservative side to hedge against inadvertent matches.

Inter-rater Reliability Measurement

The Cohen's kappa[3] of inter-rater reliability (IRR) over the population of images that we labeled is 0.73. We report the inter-rater reliability of our labeling process to demonstrate that our dataset is meaningful and consistently reproducible. While differentiating between AFOs and CAFOs using satellite data requires some judgement, a Cohen's kappa score of 0.73 (with an agreement percentage of 95.96%) shows that our labelers tend to agree on their labels. Note that these metrics are at the image level, not at the facility or building level.

Our inter-rater reliability measurement only captures the degree of agreement on one stage of our labeling process. Our labeling process includes two additional layers of review at which false positives could be further filtered out:

1. Construction date annotation by a human
2. Animal type annotation, either by a strong match to a cattle permit or a human label

We only report facilities that are confirmed as CAFOs by all three stages of the process: the initial labeling, the construction dating, and the animal typing. The consistency of the overall process is likely higher as it depends less on the judgement of any one human.

We measured the IRR of our labeling process by sampling from the labeled image pool and assigning each image to two different labelers out of a pool of four labelers. We stratified our sample into four strata:

1. Images with high model confidence labeled "no CAFO" (10056 images)
2. Images with low model confidence or no detection labeled "no CAFO" (34043 images)
3. Images initially labeled "CAFO," but later marked "no CAFO" after additional rounds of review (640 images)
4. Images labeled "CAFO" (3055 images)

200 images in each stratum were labeled by two labelers, except for the third stratum, where we labeled 174 images (at the time of sampling, only 174 images were in this category). This gave us a pool of 774 images, each of which has been labeled twice. We then sampled images with replacement from this pool. We sampled each strata in proportion to its prevalence in the true population, resulting in a set of images that matched the composition of our overall population. We calculated the Cohen's kappa on this oversampled set, resulting in a final score of 0.73.

Supplemental Figures & Tables

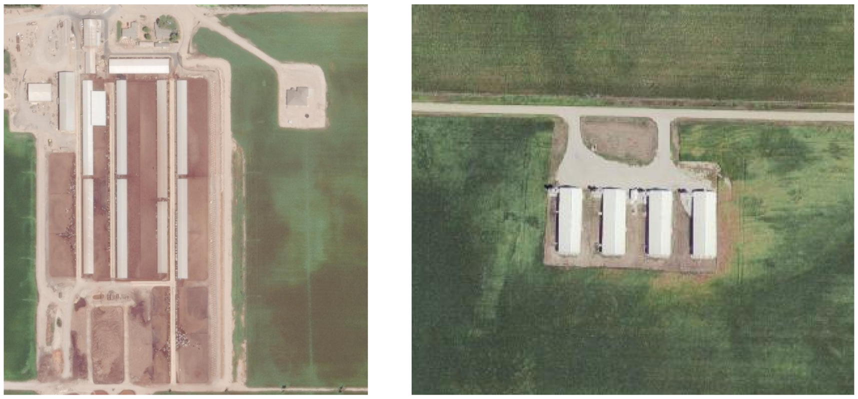

Supplementary Figure S1: On the left, a hog CAFO in Iowa; on the right, a cattle CAFO in Calfornia. Note that much of the area that houses animals is not covered, and the shape and configuration of sheds varies. However, the CAFOs continue to bear important resemblances (large areas of white roofing) that makes them comparable.

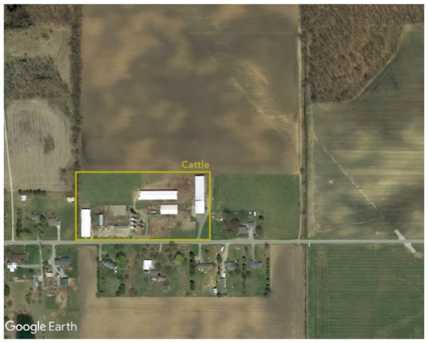

(a) mTurk Labeling Instructions, Image 1

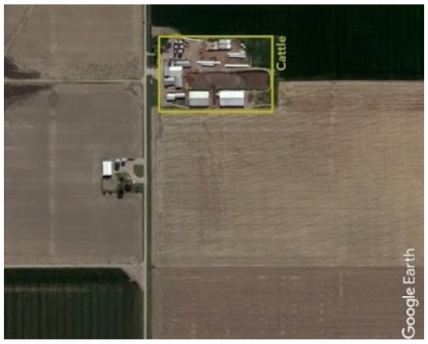

(b) mTurk Labeling Instructions, Image 2

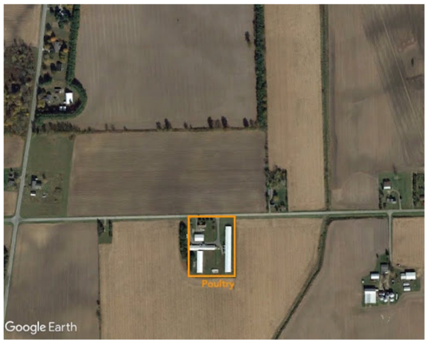

(c) mTurk Labeling Instructions, Image 3

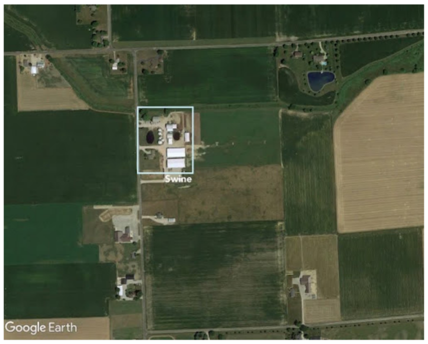

(d) mTurk Labeling Instructions, Image 4

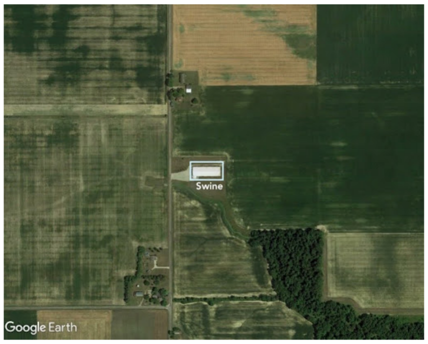

(e) mTurk Labeling Instructions, Image 5

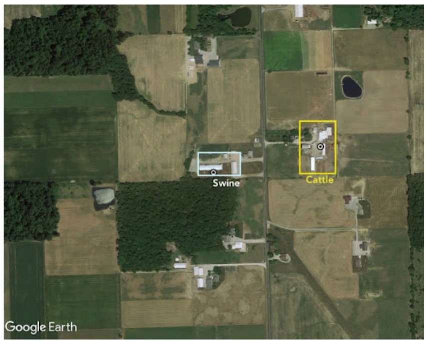

(f) mTurk Labeling Instructions, Image 6

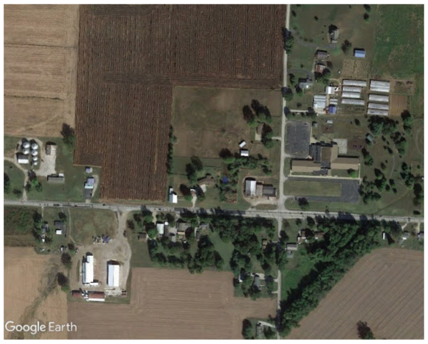

(g) mTurk Labeling Instructions, Image 7

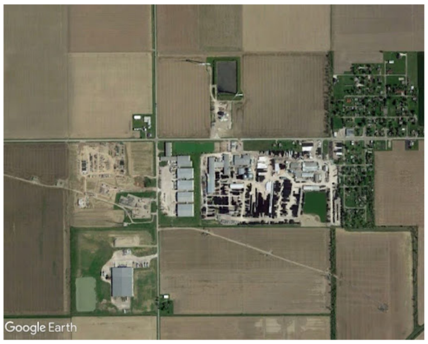

(h) mTurk Labeling Instructions, Image 8

Supplementary Figure S2: Images provided as examples within the MTurk labeling instructions.

bucket	name	unlabeled	labeled	prevalence	positive	total
high confidence & permit	completed	1	20598	0.147441	3037	20599
low confidence	2016 NAIP	9537	1623	0.001848	3	11160
low confidence	Kern	1302	1435	0.000697	1	2737
no detection	2016 NAIP	61708	1450	0.000690	1	63158
low confidence	New	6381	285	0.003509	1	6666
low confidence	Merced	233	475	0.002105	1	708
low confidence	Siskiyou	0	1387	0.000721	1	1387
low confidence	Riverside	1080	1002	0.000000	0	2082
no detection	Riverside	17292	501	0.000000	0	17793
no detection	San Bernardino	55074	1013	0.000000	0	56087
no detection	New	48478	926	0.000000	0	49404
no detection	Med 3	25240	2882	0.000000	0	28122
no detection	Med 2	24032	732	0.000000	0	24764
low confidence	San Joaquin	803	288	0.000000	0	1091
no detection	Kern	18264	1364	0.000000	0	19628
no detection	Siskiyou	17876	1595	0.000000	0	19471
no detection	Med 1	12580	437	0.000000	0	13017
low confidence	Med 1	1761	554	0.000000	0	2315
low confidence	Non-Med	8447	242	0.000000	0	8689
low confidence	Stanislaus	386	217	0.000000	0	603
low confidence	Med 2	4241	1244	0.000000	0	5485
low confidence	Med 3	1700	3747	0.000000	0	5447
no detection	Merced	2372	572	0.000000	0	2944
low confidence	San Bernardino	2399	504	0.000000	0	2903
no detection	San Joaquin	1625	298	0.000000	0	1923
no detection	Stanislaus	2132	317	0.000000	0	2449
no detection	Non-Med	106682	1835	0.000000	0	108517

Supplementary Table S1: Sampling details for estimating the number of false negative facilities missed by our CAFO detection procedure, including the total number of images in the population in each strata, the number of sampled images, the number of images found to contain a CAFO (positives), and estimated within-strata prevalence based on these labels. Strata are cut by model confidence and county groups detailed in Supplemental Table S5. The first stratum (high confidence & permit) includes everything we labeled exhaustively: high confidence detections, permits, and images adjacent to positive labels from the first two categories.

Owner 1	Owner 2
MARTINS, ROBERT & MARIA V H/W	MARTINS, ROBERTO M & MARIA H/W
BRAZIL, ROBERT E REV TRUST 50	BRAZIL, TONY J & VIRGINIA L LIV TRUST
MATTOS BROTHERS DAIRY L P	MATTOS FAMILY LIMITED PARTNERSHIP
SOZINHO FAMILY TRUST	SOZINHO, JOE S & MARY FAMILY TRUST
LEYENDEKKER GERBEN F & PAULINE V (CO-TRS)	LEYENDEKKER GERBEN F & PAULINE V(TRS)
GIOLETTI DEVIN R TR ET AL	GIOLETTI ROBERT G & ELOISE A TRS
LEYENDEKKER HENDRICK & GERALDINE (CO TRS)	LEYENDEKKER NICKOLAS & CHERYL (CO-TRS)
QUARESMA DARLENE E TR	QUARESMA SUSANNE M & RAYMOND M
MAHRT FAMILY TRUST OF 2008 ETAL	MAHRT STEVEN P
PARENTE REAL ESTATE INVESTMENT MGMNT	PARENTE REAL ESTATE INVESTMENT MNGMN
MATOS JOSE DOS ANJOS & JOVINA MARIA TRUSTEES	MATOS LARRY A TRUSTEE
GIOLETTI JUSTIN G TR ET AL	GIOLETTI SHARON L TR ET AL
DE MELO EDDIE/MARIA E	DEMELO MANUAL CABRAL/MARIA LEOPOLDINA
ORNELLAS, LEROY T & JENNIE T ETAL	ORNELLAS, MARY S TR
MACHADO DAIRY & FARMING COMPANY INC	MACHADO MARVIN JR & MARY
FARIA FAMILY I LIMITED PARTNERSHIP	FARIA FARMS INC
ASTIASUAIN DANIEL M & SUSAN J TRUSTEES	ASTIASUAIN MARTIN W & HELEN M LIFE EST
MENDONCA TONY & MARJORIE	TONY S MENDONCA & SONS A GP
LEYENDEKKER FRED J & KELSEY R(TRS)REV TR)	LEYENDEKKER GERBEN F & PAULINE V (CO-TRS)
FAGUNDES FAGUNDES FAGUNDES	FAGUNDES JAMES & GLENDA TRUSTEE
SOZINHO, JOE S & MARY FAMILY TRUST	SOZINHO, JOE S & MARY M FAMILY TRUST
MATTOS BROTHERS DAIRY L P	MATTOS FAMILY LIMITED PTP
GIOLETTI DEVIN R TR ET AL	SOUZA MANUEL S JR & LYNETTE L TRS
MATTOS FAMILY LIMITED PARTNERSHIP	MATTOS FAMILY LIMITED PTP
MACHADO DAIRY & FARMING COMPANY INC	MACHADO MARVIN B
FISCALINI FARMS	FISCALINI PROPERTIES LP
CHARLES F CABRAL BYPASS TRUST/ETAL	DEMELO MANUAL CABRAL/MARIA LEOPOLDINA
MACHADO MARVIN B	MACHADO MARVIN JR & MARY
SIMOES JOE M & MARIE I	SIMOES JOSEPH & MICHELLE
OTT ELIZABETH A TR	OTT PAULA R ET AL TRS
LUIS BERNARDETE A TRUSTEE	LUIS MANUEL S & BERNARDETE A TRUSTEES
LEYENDEKKER FRED J & KELSEY R(TRS)REV TR)	LEYENDEKKER GERBEN F & PAULINE V(TRS)
GENASCI EDWIN J TR ET AL	GENASCI RANCH
VISSER GARY L & MARTHA J TRUSTEES	VISSER GERRIT & SONS
MELO ALVIN S & LANETTE E	MELO CHERYL TR
BRADY TONY J JR & ELIZABETH M	BRADY TONY JOHN & MARY LUCY TRUSTEES
MATHERON DUANE L & BARBARA J	MATHERON RANCH DALE & DUANE A PTNRSHIP
ZYLSTRA JOHN A	ZYLSTRA NINA F ET AL TRS
DE GROOT LOUIS J & KAREN L (TRS)(L&K G TR	DE GROOT WILFRED P & SUZANNE M (TRS)
MMD MORRIS PARTNERS LP	MMJ MORRIS PARTNERS LP
SOZINHO FAMILY TRUST	SOZINHO, JOE S & MARY M FAMILY TRUST
GEMPERELE BROS A PARTNERSHIP	GEMPERLE BROS
PAULO REVOCABLE TRUST	PAULO, DONNA M
MACHADO DANIEL & DIANE	MACHADO JOHN B III & JOYCE L (TRS)(FAM TR
ROCHA JOE O FAMILY LP	ROCHA JOSEPH O CO-TRUSTEE
A. AND B. OOSTDAM FAMILY TRUST	JOHN P. OOSTDAM

Supplementary Table S2: A list of all parcel owners we consider to be related even though they do not meet our criteria of 0.7 tf-idf and 0.6 fuzzy matching score.

Owner 1	Owner 2	Fuzzy Weight
	ALBERTO DAIRY LP	0
ANZA ESTATE LLC	WHITEGOLD VENTURES LP	90
ANZA ESTATE LLC	IHC JURUPA LLC	140
5 MILE RANCH LLC	MC CLAIN BRODIE & ALLISON J	190
102-998509-0000 - I.D. NUMBER	AIROSO JOE & DIANE	240
1455 NORTH WARREN ROAD	HEIN HETTINGA	290
ALEGRE LINDA D TR	ORNELLAS LEROY & JENNIE TR	340
BACON-GADEYNE TUESDAY ANNE	GARCIA JESSIE & SANDRA	390
A K CORAL CAY TRUST 07/27/01	HARINGA, RUDY TRUST 2/5/10	440
ACKERMAN DEBRA E TR	AGRESTI DEBRA MARIE TR ET AL	490
LUIS BERNARDETE A TRUSTEE	OLIVEIRA MARIA TRUSTEE	540
BORBA LUIS L TRS ET AL	SEQUEIRA DOUGLAS L ET AL	590
CITY OF ONTARIO	JONGS ONTARIO LLC	640
BRINDEIRO & DANBOM	BRINDEIRO YVONNA	690
ROBERT SEIFERT PROPERTIES LLC	SEIFERT PROPERITES LLC	740
MACHADO MARVIN B	MACHADO MARVIN JR & MARY	790
ACKERMAN DEBRA E TR	ACKERMAN DEBRA E TR ET AL	840
TERRA LINDA DAIRY	TERRA LINDA DAIRY GP	880
AIROSO JOE & DIANE	AIROSO JOE G & DIANE	940

Supplementary Table S3: Examples of strings and their fuzzy matching scores.

Owner 1	Owner 2	TF-IDF Weight
BADER, RONALD G TR ETAL	DA SILVA, JOE & ANA TR	49
DUNNIGAN RANCH LLC	JONGS ONTARIO LLC	99
DEWIT DAVID ETAL	RAUSSER CHARLOTTE L TR ETAL	142
SEQUEIRA DOUGLAS L ET AL	VITORINO DUARTE JOSE ET AL	195
SANTOS RICHARD M TR ET AL	VITORINO JOHN M JR ET AL	244
BORBA LUIS L TRS ET AL	SILVA MANUEL M TR ET AL	295
R4 Ranches	Renner Ranches Inc	325
GEMPERLE BROTHERS	GERMANN BROTHERS DAIRY	397
HETTINGA FARMS	HETTINGA STEVE & ARLENE ANN	447
BROWN'S DAIRY A PTP	MELLO, J D DAIRY A PTP	498
VAN EXEL DAIRY PTP	VAN EXEL, HENRY & CAROLYN TR	548
BORBA FAMILY RANCHES LP	R&R RANCHES L L C	576
BRASIL EDWIN & LILIAN	BRASIL JOAO E & LILIAN M	627
FISCALINI FARMS	FISCALINI PROPERTIES LP	672
BRASIL JOHN H & MARIA G TRS	BRASIL JOHN H ET AL TRS	727
MACHADO MARVIN B	MACHADO MARVIN JR & MARY	791
TERRA LINDA DAIRY	TERRA LINDA DAIRY GP	840
DROOGH DAIRY L P	DROOGH DAIRY LP	899
NISSEN, DANA & GRACE TR ETAL	NISSEN, DANA P & GRACE TR	946

Supplementary Table S4: Examples of strings and their tf-idf scores.

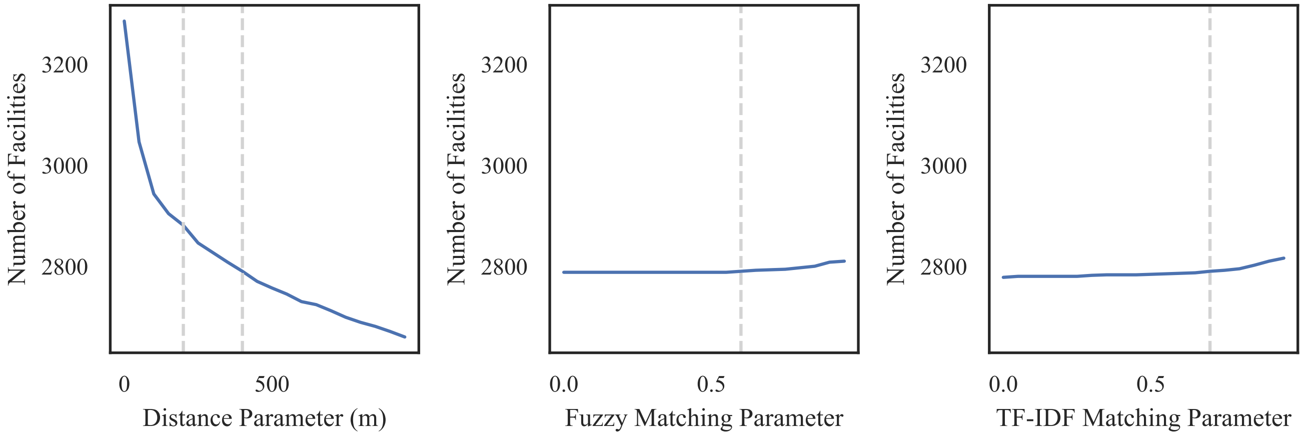

Supplementary Figure S3: Sensitivity of the number of detected facilities to the three parameters of our building clustering procedure. The first is the distance between buildings on different parcels and the two others are measures of string similarity of parcel owner names: fuzzy matching and tf-idf score. These plots show the relationship between the number of building clusters and the value of these parameter thresholds. Each plot holds two parameters fixed at their final threshold values and varies the third, with a grey dashed line illustrating the chosen threshold value: a distance of 200 meters without ownership data and 400 meters with, fuzzy matching score of 0.6, and TF-IDF score of 0.7.

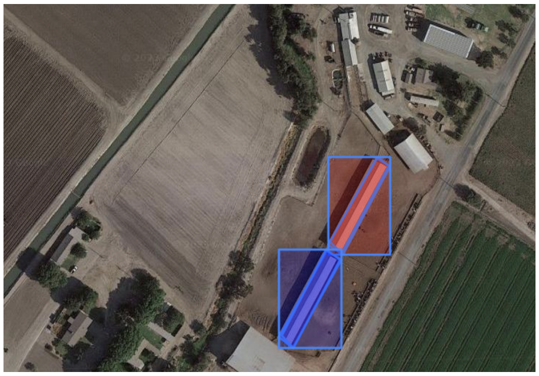

Supplementary Figure S4: Example of an edge case of errant parcel geocoding. These two buildings are clearly part of the same lot and under common operation. They are just three meters apart. The building in blue was geocoded to a parcel owned by 'Nicoletti Cynthia Trustee'; the building in red was geocoded to a parcel owned by 'Poso Canal Co A Corp'. Note the canal visible in the image. This error is likely a result of irregular parcel boundaries around this canal; we observe similar problems near other bodies of water and railroads.

County Group	Counties
Group 1	Colusa, Los Angeles, Yuba
Group 2	Butte, Marin, Placer, Sacramento, San Diego, Ventura
Group 3	Fresno, Kings, Madera, Monterey, San Benito, San Luis Obispo, Tulare
Group 4	Alameda, Amador, El Dorado, Mendocino, Napa, Nevada, Orange, San Francisco, Santa Clara, Sutter
Group 5	Contra Costa, Glenn, Humboldt, Imperial, Lake, Mariposa, San Mateo, Santa Cruz, Solano, Sonoma, Tuolumne
Group 6	Alpine, Calaveras, Del Norte, Inyo, Lassen, Modoc, Mono, Plumas, Santa Barbara, Shasta, Sierra, Tehama, Trinity, Yolo

Supplementary Table S5: County Sampling groups, counties grouped together were sampled as if they were one county during our sampling procedure.

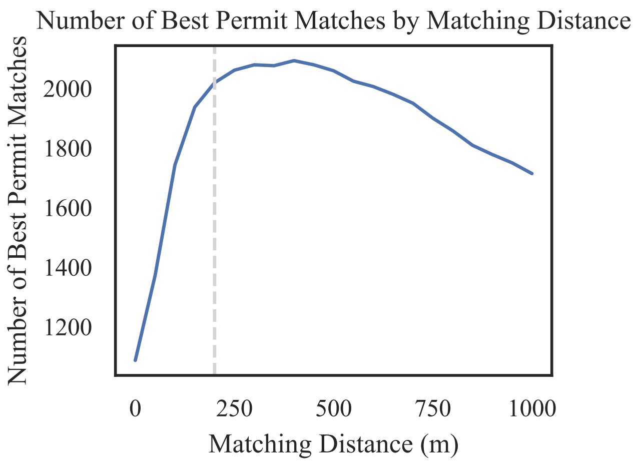

Supplementary Figure S5: Relationship between the number of permits that are a best match to one facility and the distance threshold on the matching criteria. The dashed gray line indicates the chosen threshold of 200m.

	Permit within 1km		
Animal Type	Yes	No	Total
Cattle	79	1498	1577
Poultry	58	256	314
Dairy	6	177	183
Swine	8	21	29
Unknown	2	7	9
Two Or More	2	3	5
Sheep	2	1	3
Goats	0	1	1
Total	157	1964	2121

Supplementary Table S6: Number of CAFOs by animal type, distinguishing between facilities with a permit registered within 1km of the facility and facilities without a permit registered within 1km.

References

[1] Jocher, G. *et al.* ultralytics/yolov3: v9.6.0 - YOLOv5 v6.0 release compatibility update for YOLOv3, https://doi.org/10.5281/zenodo.5701405 (2021).

[2] Kingma, D. P. & Ba, J. Adam: A Method for Stochastic Optimization (2017). https://arxiv.org/abs/1412.6980. 1412.6980.

[3] Cohen, J. A coefficient of agreement for nominal scales. *Educational and Psychological Measurement* **20**, 37–46, https://doi.org/10.1177/001316446002000104 (1960).
